# 4-(4-Fluoro­phen­oxy)benzoic acid

**DOI:** 10.1107/S1600536809028232

**Published:** 2009-07-25

**Authors:** Hoong-Kun Fun, Jia Hao Goh, Sankappa Rai, Prakash Shetty, Arun M. Isloor

**Affiliations:** a X-ray Crystallography Unit, School of Physics, Universiti Sains Malaysia, 11800 USM, Penang, Malaysia; b Syngene International Ltd, Biocon Park, Plot Nos. 2 & 3, Bommasandra 4th Phase, Jigani Link Road, Bangalore 560 100, India; c Department of Printing, Manipal Institute of Technology, Manipal 576 104, India; d Department of Chemistry, National Institute of Technology-Karnataka, Surathkal, Mangalore 575 025, India

## Abstract

In the title compound, C_13_H_9_FO_3_, the dihedral angle between the two benzene rings is 70.99 (5)°. In the crystal structure, mol­ecules are linked into dimers by centrosymmetric O—H⋯O inter­actions, generating *R*_2_^2^(8) ring motifs. These dimers are linked into a two-dimensional array, parallel to the *ab* plane, by two different C—H⋯O inter­actions. A weak C—H⋯π inter­actions is also present.

## Related literature

For general background to and applications of phen­oxy benzoic acid derivatives, see: Forster *et al.* (1989 ▶); Holla *et al.* (2003 ▶); Ramu *et al.* (2000 ▶). For the stability of the temperature controller used for the data collection, see: Cosier & Glazer (1986 ▶).
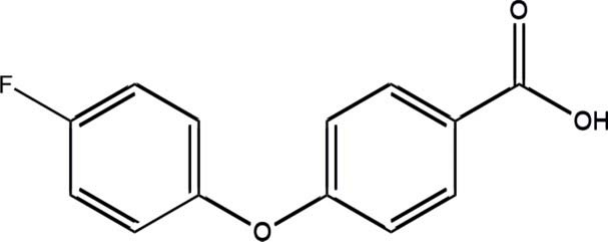


## Experimental

### 

#### Crystal data

C_13_H_9_FO_3_
*M* *_r_* = 232.20
Triclinic, 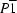

*a* = 5.8850 (1) Å
*b* = 7.8526 (2) Å
*c* = 12.0250 (2) Å
α = 91.803 (1)°
β = 96.321 (1)°
γ = 106.027 (1)°
*V* = 529.75 (2) Å^3^
*Z* = 2
Mo *K*α radiation
μ = 0.12 mm^−1^
*T* = 100 K
0.38 × 0.22 × 0.14 mm

#### Data collection

Bruker SMART APEXII CCD area-detector diffractometer
Absorption correction: multi-scan (**SADABS**; Bruker, 2005 ▶) *T*_min_ = 0.958, *T*_max_ = 0.984
17124 measured reflections
4049 independent reflections
3329 reflections with *I* > 2σ(*I*)
*R*_int_ = 0.023

#### Refinement

*R*[*F* ^2^ > 2σ(*F* ^2^)] = 0.045
*wR*(*F* ^2^) = 0.123
*S* = 1.04
4049 reflections
190 parameters
All H-atom parameters refined
Δρ_max_ = 0.48 e Å^−3^
Δρ_min_ = −0.26 e Å^−3^

### 

Data collection: *APEX2* (Bruker, 2005 ▶); cell refinement: *SAINT* (Bruker, 2005 ▶); data reduction: *SAINT*; program(s) used to solve structure: *SHELXTL* (Sheldrick, 2008 ▶); program(s) used to refine structure: *SHELXTL*; molecular graphics: *SHELXTL*; software used to prepare material for publication: *SHELXTL* and *PLATON* (Spek, 2009 ▶).

## Supplementary Material

Crystal structure: contains datablocks global, I. DOI: 10.1107/S1600536809028232/tk2506sup1.cif

Structure factors: contains datablocks I. DOI: 10.1107/S1600536809028232/tk2506Isup2.hkl

Additional supplementary materials:  crystallographic information; 3D view; checkCIF report

## Figures and Tables

**Table 1:** Hydrogen-bond geometry (Å, °)

*D*—H⋯*A*	*D*—H	H⋯*A*	*D*⋯*A*	*D*—H⋯*A*
O2—H1*O*2⋯O3^i^	0.92 (2)	1.70 (2)	2.6204 (11)	175 (2)
C5—H5*A*⋯O3^ii^	0.980 (15)	2.403 (15)	3.3573 (12)	164.3 (14)
C9—H9*A*⋯O2^iii^	0.969 (17)	2.588 (16)	3.3519 (13)	135.9 (11)
C2—H2*A*⋯*Cg*2^iv^	0.981 (15)	2.928 (16)	3.9014 (10)	172.1 (13)

Symmetry codes: (i) 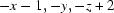; (ii) 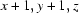; (iii) 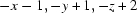; (iv) 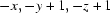. *Cg*1 is the centroid of the C1–C6 benzene ring.

**Fig. 1.**
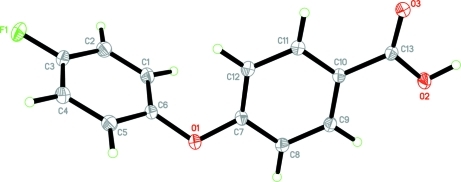
The molecular structure of (I), showing 50% probability displacement ellipsoids and the atom-numbering scheme.

**Fig. 2.**
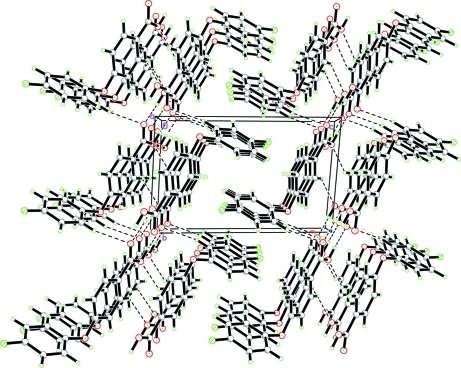
2-D arrays parallel to the ab plane, viewed along the a axis. Intermolecular interactions are shown as dashed lines.

## supplementary crystallographic information

### Comment

Phenoxy benzoic acids and its derivatives are known for their herbicidal and plant growth-regulating activities (Forster *et al.*,  1989). These compounds are also used in the synthesis of various thiadiazoles and oxadiazole derivatives which show excellent anti-bacterial activity (Holla *et al.*, 2003).The title compound, (I), which is used for peripheral neuropathic pain treatment, is a potent blocker of neuronal voltage-gated sodium channels that interacts selectively with inactivated states as opposed to resting states of the channels (Ramu *et al.*,  2000).

In (I), Fig. 1, the two benzene rings are inclined to one another, with dihedral angle of 70.99 (5)°. In the crystal structure (Fig. 2), the molecules are linked into dimers by centrosymmetric O2—H1O2···O3 interactions (Table 1) to generate *R*^2^_2_(8) ring motifs. These dimers are linked into a 2-D array, parallel to the *ab* plane, by intermolecular C—H···O interactions (Table 1). The crystal structure is further stabilized by weak C2—H2A···Cg2 (Table 1) and π···π interactions involving the C1-C6 benzene rings (centroid Cg1) [Cg1···Cg1 = 3.6427 (6)°; symmetry code: 1-x, 2-y, 1-z].

### Experimental

4-Bromo-methylbenzoate (0.760 g, 3.57 mmol), sodium carbonate (0.750 g, 7.00 mmol), and tetrakis(triphenylphosphine)palladium(0) (0.400 g, 0.350 mmol) were added to a stirred solution of 4-fluorobenzene boronic acid (0.500 g, 3.57 mmol) in toluene and water (1:1) (20 ml). The reaction mixture was heated at reflux for 8 h; TLC indicated completion of reaction. Sodium hydroxide (0.284 g, 7.00 mmol) was added and stirring was continued for further 1 h. Mass analysis of crude reaction mixture shows the formation of desired compound. The reaction mixture was acidified to pH 3, extracted with ethylacetate and dried. The concentrated residue was purified by column chromatography to yield the pure product, which was recrystallized using hot dichloromethane to yield single crystals. The yield was 0.400 g, 50 %. *M.p.* 448-450 K.

### Refinement

All the H atoms were located from difference Fourier map and allowed to refine freely [range of C—H = 0.959 (15) - 0.981 (15) Å].

### Crystal data

**Table:**

C_13_H_9_FO_3_	*Z* = 2
*M**_r_* = 232.20	*F*(000) = 240
Triclinic, *P*1	*D*_x_ = 1.456 Mg m^−^^3^
Hall symbol: -P 1	Mo *K*α radiation, λ = 0.71073 Å
*a* = 5.8850 (1) Å	Cell parameters from 7298 reflections
*b* = 7.8526 (2) Å	θ = 2.7–33.2°
*c* = 12.0250 (2) Å	µ = 0.12 mm^−^^1^
α = 91.803 (1)°	*T* = 100 K
β = 96.321 (1)°	Block, colourless
γ = 106.027 (1)°	0.38 × 0.22 × 0.14 mm
*V* = 529.75 (2) Å^3^	

### Data collection

**Table:**

Bruker SMART APEXII CCD area-detector diffractometer	4049 independent reflections
Radiation source: fine-focus sealed tube	3329 reflections with *I* > 2σ(*I*)
graphite	*R*_int_ = 0.023
φ and ω scans	θ_max_ = 33.3°, θ_min_ = 2.7°
Absorption correction: multi-scan (*SADABS*; Bruker, 2005)	*h* = −9→9
*T*_min_ = 0.958, *T*_max_ = 0.984	*k* = −12→12
17124 measured reflections	*l* = −18→18

### Refinement

**Table:**

Refinement on *F*^2^	Primary atom site location: structure-invariant direct methods
Least-squares matrix: full	Secondary atom site location: difference Fourier map
*R*[*F*^2^ > 2σ(*F*^2^)] = 0.045	Hydrogen site location: inferred from neighbouring sites
*wR*(*F*^2^) = 0.123	All H-atom parameters refined
*S* = 1.04	*w* = 1/[σ^2^(*F*_o_^2^) + (0.0614*P*)^2^ + 0.1453*P*] where *P* = (*F*_o_^2^ + 2*F*_c_^2^)/3
4049 reflections	(Δ/σ)_max_ < 0.001
190 parameters	Δρ_max_ = 0.48 e Å^−^^3^
0 restraints	Δρ_min_ = −0.26 e Å^−^^3^

### Special details

**Table:**

Experimental. The crystal was placed in the cold stream of an Oxford Cyrosystems Cobra open-flow nitrogen cryostat (Cosier & Glazer, 1986) operating at 100.0 (1)K.
Geometry. All esds (except the esd in the dihedral angle between two l.s. planes) are estimated using the full covariance matrix. The cell esds are taken into account individually in the estimation of esds in distances, angles and torsion angles; correlations between esds in cell parameters are only used when they are defined by crystal symmetry. An approximate (isotropic) treatment of cell esds is used for estimating esds involving l.s. planes.
Refinement. Refinement of F^2^ against ALL reflections. The weighted R-factor wR and goodness of fit S are based on F^2^, conventional R-factors R are based on F, with F set to zero for negative F^2^. The threshold expression of F^2^ > 2sigma(F^2^) is used only for calculating R-factors(gt) etc. and is not relevant to the choice of reflections for refinement. R-factors based on F^2^ are statistically about twice as large as those based on F, and R- factors based on ALL data will be even larger.

### Fractional atomic coordinates and isotropic or equivalent isotropic displacement parameters (Å^2^)

**Table:**

	*x*	*y*	*z*	*U*_iso_*/*U*_eq_	
F1	0.72786 (13)	0.78346 (9)	0.39118 (6)	0.02925 (17)	
O1	0.21383 (14)	0.80788 (9)	0.74196 (6)	0.02239 (16)	
O2	−0.50311 (14)	0.22204 (10)	0.97542 (7)	0.02221 (17)	
O3	−0.26343 (13)	0.05949 (9)	0.93084 (6)	0.01906 (15)	
C1	0.22933 (18)	0.70062 (13)	0.55272 (9)	0.01978 (19)	
C2	0.36012 (18)	0.69543 (13)	0.46395 (8)	0.02022 (19)	
C3	0.59978 (18)	0.78592 (13)	0.47858 (8)	0.01926 (19)	
C4	0.71427 (18)	0.88095 (13)	0.57691 (9)	0.02004 (19)	
C5	0.58253 (18)	0.88423 (13)	0.66581 (8)	0.01836 (18)	
C6	0.34309 (17)	0.79342 (12)	0.65322 (8)	0.01679 (17)	
C7	0.08790 (17)	0.65375 (12)	0.78461 (8)	0.01647 (17)	
C8	−0.09821 (18)	0.66886 (12)	0.84280 (8)	0.01712 (18)	
C9	−0.23330 (17)	0.52057 (12)	0.88889 (8)	0.01649 (17)	
C10	−0.17956 (16)	0.35800 (12)	0.87894 (7)	0.01449 (16)	
C11	0.01210 (17)	0.34637 (12)	0.82318 (8)	0.01577 (17)	
C12	0.14587 (17)	0.49364 (12)	0.77501 (8)	0.01720 (18)	
C13	−0.31845 (16)	0.20040 (12)	0.93050 (7)	0.01521 (17)	
H1O2	−0.578 (4)	0.120 (3)	1.0074 (18)	0.067 (6)*	
H1A	0.061 (3)	0.6429 (19)	0.5446 (12)	0.027 (4)*	
H2A	0.287 (3)	0.634 (2)	0.3911 (13)	0.030 (4)*	
H4A	0.883 (2)	0.9437 (18)	0.5838 (12)	0.023 (3)*	
H5A	0.653 (3)	0.9516 (19)	0.7372 (13)	0.028 (4)*	
H8A	−0.134 (3)	0.780 (2)	0.8501 (12)	0.027 (4)*	
H9A	−0.366 (3)	0.5275 (19)	0.9281 (12)	0.025 (3)*	
H11A	0.051 (2)	0.2356 (18)	0.8193 (11)	0.020 (3)*	
H12A	0.279 (3)	0.4852 (19)	0.7355 (12)	0.026 (3)*	

### Atomic displacement parameters (Å^2^)

**Table:**

	*U*^11^	*U*^22^	*U*^33^	*U*^12^	*U*^13^	*U*^23^
F1	0.0317 (4)	0.0303 (3)	0.0262 (3)	0.0042 (3)	0.0181 (3)	−0.0015 (3)
O1	0.0293 (4)	0.0139 (3)	0.0247 (4)	0.0022 (3)	0.0160 (3)	0.0027 (3)
O2	0.0236 (4)	0.0175 (3)	0.0295 (4)	0.0067 (3)	0.0162 (3)	0.0065 (3)
O3	0.0238 (4)	0.0146 (3)	0.0205 (3)	0.0054 (3)	0.0091 (3)	0.0029 (2)
C1	0.0177 (4)	0.0185 (4)	0.0211 (4)	0.0009 (3)	0.0045 (3)	0.0007 (3)
C2	0.0223 (5)	0.0193 (4)	0.0176 (4)	0.0030 (3)	0.0037 (3)	0.0002 (3)
C3	0.0217 (4)	0.0183 (4)	0.0193 (4)	0.0052 (3)	0.0099 (3)	0.0027 (3)
C4	0.0163 (4)	0.0189 (4)	0.0239 (5)	0.0025 (3)	0.0052 (3)	0.0006 (3)
C5	0.0196 (4)	0.0166 (4)	0.0179 (4)	0.0032 (3)	0.0030 (3)	0.0008 (3)
C6	0.0200 (4)	0.0132 (4)	0.0178 (4)	0.0033 (3)	0.0079 (3)	0.0031 (3)
C7	0.0186 (4)	0.0142 (4)	0.0156 (4)	0.0015 (3)	0.0054 (3)	0.0019 (3)
C8	0.0211 (4)	0.0148 (4)	0.0172 (4)	0.0061 (3)	0.0067 (3)	0.0028 (3)
C9	0.0176 (4)	0.0171 (4)	0.0164 (4)	0.0057 (3)	0.0061 (3)	0.0027 (3)
C10	0.0159 (4)	0.0143 (4)	0.0131 (4)	0.0033 (3)	0.0038 (3)	0.0019 (3)
C11	0.0167 (4)	0.0147 (4)	0.0161 (4)	0.0039 (3)	0.0043 (3)	0.0012 (3)
C12	0.0166 (4)	0.0166 (4)	0.0188 (4)	0.0035 (3)	0.0066 (3)	0.0013 (3)
C13	0.0169 (4)	0.0153 (4)	0.0131 (4)	0.0032 (3)	0.0040 (3)	0.0008 (3)

### Geometric parameters (Å, °)

**Table:**

F1—C3	1.3617 (11)	C5—C6	1.3814 (14)
O1—C7	1.3801 (11)	C5—H5A	0.980 (15)
O1—C6	1.3961 (11)	C7—C8	1.3923 (13)
O2—C13	1.3142 (11)	C7—C12	1.3953 (13)
O2—H1O2	0.92 (2)	C8—C9	1.3863 (13)
O3—C13	1.2357 (11)	C8—H8A	0.959 (15)
C1—C2	1.3889 (14)	C9—C10	1.4021 (13)
C1—C6	1.3917 (14)	C9—H9A	0.967 (14)
C1—H1A	0.960 (15)	C10—C11	1.3963 (13)
C2—C3	1.3817 (14)	C10—C13	1.4783 (13)
C2—H2A	0.981 (15)	C11—C12	1.3901 (13)
C3—C4	1.3786 (14)	C11—H11A	0.961 (13)
C4—C5	1.3916 (14)	C12—H12A	0.978 (15)
C4—H4A	0.971 (14)		
			
C7—O1—C6	118.23 (7)	O1—C7—C12	123.17 (9)
C13—O2—H1O2	109.9 (13)	C8—C7—C12	121.23 (8)
C2—C1—C6	119.42 (9)	C9—C8—C7	119.35 (9)
C2—C1—H1A	120.5 (9)	C9—C8—H8A	120.3 (9)
C6—C1—H1A	120.1 (9)	C7—C8—H8A	120.3 (9)
C3—C2—C1	118.26 (9)	C8—C9—C10	120.22 (9)
C3—C2—H2A	119.4 (9)	C8—C9—H9A	120.7 (9)
C1—C2—H2A	122.2 (9)	C10—C9—H9A	119.1 (9)
F1—C3—C4	118.50 (9)	C11—C10—C9	119.70 (8)
F1—C3—C2	118.45 (9)	C11—C10—C13	119.66 (8)
C4—C3—C2	123.04 (9)	C9—C10—C13	120.60 (8)
C3—C4—C5	118.37 (9)	C12—C11—C10	120.43 (8)
C3—C4—H4A	120.6 (8)	C12—C11—H11A	120.5 (8)
C5—C4—H4A	121.0 (8)	C10—C11—H11A	119.1 (8)
C6—C5—C4	119.50 (9)	C11—C12—C7	119.03 (9)
C6—C5—H5A	118.4 (9)	C11—C12—H12A	120.4 (9)
C4—C5—H5A	122.1 (9)	C7—C12—H12A	120.6 (9)
C5—C6—C1	121.40 (9)	O3—C13—O2	123.01 (9)
C5—C6—O1	117.85 (9)	O3—C13—C10	122.01 (8)
C1—C6—O1	120.60 (9)	O2—C13—C10	114.98 (8)
O1—C7—C8	115.55 (8)		
			
C6—C1—C2—C3	−1.01 (15)	O1—C7—C8—C9	−179.70 (9)
C1—C2—C3—F1	−179.00 (9)	C12—C7—C8—C9	−2.13 (15)
C1—C2—C3—C4	−0.10 (16)	C7—C8—C9—C10	1.33 (14)
F1—C3—C4—C5	179.56 (9)	C8—C9—C10—C11	0.55 (14)
C2—C3—C4—C5	0.66 (16)	C8—C9—C10—C13	178.31 (9)
C3—C4—C5—C6	−0.11 (15)	C9—C10—C11—C12	−1.68 (14)
C4—C5—C6—C1	−1.01 (15)	C13—C10—C11—C12	−179.47 (8)
C4—C5—C6—O1	−176.49 (9)	C10—C11—C12—C7	0.91 (14)
C2—C1—C6—C5	1.58 (15)	O1—C7—C12—C11	178.39 (9)
C2—C1—C6—O1	176.94 (9)	C8—C7—C12—C11	1.01 (15)
C7—O1—C6—C5	−125.60 (10)	C11—C10—C13—O3	3.66 (14)
C7—O1—C6—C1	58.88 (13)	C9—C10—C13—O3	−174.10 (9)
C6—O1—C7—C8	−158.42 (9)	C11—C10—C13—O2	−176.33 (8)
C6—O1—C7—C12	24.07 (14)	C9—C10—C13—O2	5.90 (13)

### Hydrogen-bond geometry (Å, °)

**Table:**

*D*—H···*A*	*D*—H	H···*A*	*D*···*A*	*D*—H···*A*
O2—H1O2···O3^i^	0.92 (2)	1.70 (2)	2.6204 (11)	175 (2)
C5—H5A···O3^ii^	0.980 (15)	2.403 (15)	3.3573 (12)	164.3 (14)
C9—H9A···O2^iii^	0.969 (17)	2.588 (16)	3.3519 (13)	135.9 (11)
C2—H2A···Cg2^iv^	0.981 (15)	2.928 (16)	3.9014 (10)	172.1 (13)

Symmetry codes: (i) −*x*−1, −*y*, −*z*+2; (ii) *x*+1, *y*+1, *z*; (iii) −*x*−1, −*y*+1, −*z*+2; (iv) −*x*, −*y*+1, −*z*+1.
